# Relationship between consanguinity and depression in a south Indian population

**DOI:** 10.4103/0019-5545.44906

**Published:** 2009

**Authors:** T. S. Sathyanarayana Rao, A. K. Prabhakar, K. S. Jagannatha Rao, K. Sambamurthy, M. R. Asha, Dushad Ram, Ananya Nanda

**Affiliations:** Department of Psychiatry, JSS University, JSS Medical College Hospital, Ramanuja Road, Mysore – 570 004, India; 1 Biochemistry and Nutrition, CFTRI, Mysore-570 020, India; 2 Department of Neuroscience, Medical university of South Carolina, Charleston, SC, 29425, USA; 3 Reiki Practitioner and Freelance writer, 788/160, 18th Cross, Ramanuja Road, Mysore, India

**Keywords:** Consanguinity, depression, marriage

## Abstract

A Pilot study was Carried out to study the association of consanguinity marriage with depression. It was observed that the consanguinity of marriage was associated with depression. The odds ratio was 5.66 (CI: 2.42-13.54). The age and sex had an association with depression. The age and sex adjusted odds ratio of consanguinity marriage was 7.66 (CI: 3.93-19.45) indicating that it is independently associated with depression.

## INTRODUCTION

Depression has a complex etiology and often results from a combination of multiple factors. The syndromal depression is linked to physical changes in the brain and to an imbalance of neurotransmitters that carries signals in brain and nerves. Some of the more common factors involved in triggering depression are: Family history, which includes genetics, trauma and stress, which includes financial problems, breaks in relationships or the death of a loved one and other life-changing events such as starting a new job, graduating from school or getting married. Further, pessimistic personality traits like low self-esteem and a negative outlook lead to higher risk of becoming depressed. Sometimes serious medical conditions like heart disease, cancer, and HIV can play a significant role in causing depression.[1–4] However, there are very limited studies interlinking consanguinity and depression. Consanguinity of marriages is prevalent in some communities in the Indian population, especially in south India. It is estimated that, at the national level prevalence of cousin marriages is 14%, the overall figure conceals the regional and religious differences. For example, in Tamil Nadu 47 percent of marriages were consanguineous compared to 25 percent in Maharashtra and less than 10 percent in northern and eastern India.[5] Bittles *et al*, in a large scale study in two cities in Karnataka, reported the prevalence of consanguineous marriage in Bangalore and Mysore to be 37% and 31% respectively.[6] Studies have shown that the consanguinity of marriages is associated with many diseases related to birth malformations due to expression of recessive defects.[7–15] The genetics of depression is still not understood.[16–18] The clinical observations indicated that the depression is very high in some communities where the consanguinity of marriages is also high.[19] However, it is still a debatable topic. In this study, we present evidence showing the association of depression with consanguineous marriages, which suggests that recessive genetic factors influence depression.

## MATERIALS AND METHODS

The study was planned with a case–control design. Proven depression cases based on DSM IV TR[20] criteria were selected from the out patient department of JSS medical college, Mysore and Vinayaka Nursing Home, Mandya during 2006-2008 after obtaining the appropriate consent. The controls were selected from consented patients from the departments of Surgery, Emergency and Orthopedics of JSS medical college, Mysore. The subjects for the study were in the age group 20-70 years of both sexes. The information on age, sex, educational status, community and consanguinity of marriages was obtained from each subject and included in the study. Univariate analysis was carried out with consanguinity and other factors to study the association. Also estimated the odds ratios for factors, which had association with depression. The bivariate logistic regression analysis was adopted to study the independent association of consanguinity of marriage with depression.

## RESULTS

The mean age of subjects included in the study was 39.04 (standard deviation 16.93), the mean age of cases was 33.64 and controls was 45.51. The females constituted 36.5 percent with 37.1 percent in controls compared to 43.9 in cases. The data indicated that the consanguinity was found to be associated with the depression. The odds ratio was 5.66 with confidence interval 2.42-13.54 [Table 1]. It was observed that in younger age groups, the OR was 6.43 compared to persons above age 50 years. The females had higher risk of depression compared to males (OR 2.34). The univariate analysis with other factors with depression indicated that age and sex were associated with depression. The educational status and community had no association with consanguinity [Table 2].

**Table 1:** The Odds ratio with confidence interval of consanguinity marriage with depression

Consanguinity↓	Depression	Control	Total	Odds ratio
No	64 (40.4)	74 (53.0)	138 (100.0)	1.00
Yes	44 (83.0)	9 (17.0)	53 (100/0)	5.66 (2.42-13.54)
Total	180 (56.5)	83 (43.5)	191 (100/0)

Number in parenthesis represents percentages (*P*<.05)

**Table 2:** The Odds ratio with confidence interval of socio demographic factors with depression

Factors	Classification		Case	Controls	*P* value	Odds ratio
1.	Sex	Males	59	62	*P*<0.05	1.00
		Females	49	22		2.34 (1.21-4.55)
2.	Age distribution (In years)	50	14	36	*P*<0.05	1.00
		40-49	22	16		3.54 (1.33-9.57)
		30-39	30	12		6.43 (2.37-17.88)
		20-29	32	16		5.14 (2.00-13.43)
		< 20	10	4		6.43 (6.50-29.74)
3.	Education	Not literate	37	37	*P*>0.05
		Primary School	5	9
		Middle School	16	15
		High School	27	16
		PUC/ diploma	14	6
		Graduate	9	2
4	Community	Gowda	53	35	*P*>0.05
		Lingayat	15	18
		Others	40	31

The multivariate analysis with bivariate logistic regression analysis was applied to study the independent association of consanguinity of marriages with depression after removing the effect of age and sex. It was observed that the odds ratio of consanguinity was. 7.32 (3.93-19.45). The classificatory analysis indicated that with the three variables, it is possible to classify correctly 69.1 percent of subjects [Table 3]. The consanguity marriage alone could classify 61.9% correctly [Table 4].

**Table 3:** Classification of subjects with bivariate regression analysis of consanguinity of marriage, age and sex with depression

Observed/↓ Predicted→	Yes	No	Percentage with correct
Yes	85	23	78.7
No	36	47	56.5
Over all percentage			69.1

**Table 4:** Classification of subjects with bivariate regression analysis of consanguinity of marriage with depression

Observed/↓ Predicted→	Yes	No	Percentage with correct
Yes	44	64	40.7
No	9	74	89.2
Over all percentage			61.9

## DISCUSSION

Depression is a serious medical condition that leads to social problems as well as loss of productivity. It is generally under-diagnosed and leads to severe suffering among the subjects. The findings in this study suggest that the consanguinity of marriage has higher risk associated with depression. This is a pilot study that needs to be confirmed and extended but this investigation provided lead data on the positive relationship between consanguinity and depression. We further extending the study to further test this hypothesis with a larger sample after excluding other known factors related to depression. The high levels of depression in consanguineous marriages may be genetically driven. If so, the identification of recessive mediators of depression can lead to a number of useful mechanistic, diagnostic and therapeutic possibilities. This type of recessive gene analysis is generally difficult even with twin studies, as twins generally share traits, but they are not necessarily a product of homozygosis due to marriages between relatives. Alternatively, the social structure that leads to consanguinity can create a number of situations that lead to depression. In either event, the understanding of the higher risk of depression in consanguineous marriages can help in the prevention of or management of this serious condition.
